# Translation, cultural adaptation and evaluation of the psychometric properties of the Hamilton Anxiety Scale among a sample of Portuguese adult patients with mental health disorders

**DOI:** 10.1186/s12888-023-05010-5

**Published:** 2023-07-19

**Authors:** Eugénia Raquel Pinheiro dos Santos, Joana Catarina Ferreira Coelho, Isilda Ribeiro, Francisco Sampaio

**Affiliations:** 1 Surgery Department, Hospital Center of Vila Nova de Gaia/Espinho, EPE, Vila Nova de Gaia, 4434-502 Portugal; 2 Portuguese Red Cross Northern Health School, Rua da Cruz Vermelha Cidacos-Apartado 1002, Oliveira de Azeméis, 3720-126 Portugal; 3 CINTESIS@RISE, Nursing School of Porto (ESEP), Rua Dr Plácido da Costa, Porto, 4200-450 Portugal; 4 Nursing School of Porto, Rua Dr. António Bernardino de Almeida, 830, 844, 856, Porto, 4200-072 Portugal

**Keywords:** Anxiety, Hamilton Anxiety Scale, Psychiatry, Psychometrics

## Abstract

**Background:**

Only a few anxiety assessment tools that nurses may administer are validated forthe Portuguese population exist in the literature. Thus, this study aimed to translate and culturally adapt the Hamilton Anxiety Scale for the Portuguese population and assess its psychometric properties in a sample of adult people with mental health disorders.

**Methods:**

This psychometric study uses a convenience sample of adult patients with mental health disorders.

**Results:**

The confirmatory factor analysis confirmed the two factors of the original version of the tool. The internal consistency (Cronbach’s alpha) was high, at .92, as well as the inter-rater reliability (intraclass correlation coefficient) (.91).

**Conclusions:**

The validity and reliability of the instrument are supported. However, the Hamilton Anxiety Scale should be used cautiously in the Portuguese population because the correlation with the “Anxiety State” subscale of the State-Trait Anxiety Inventory is not statistically significant.

## Background

Anxiety has been defined as a feeling of unease that may translate into physiological, motor, and cognitive manifestations. These manifestations may be associated with events or situations of a temporary nature (state anxiety) or constitute a stable and permanent way of reacting to problems (trait anxiety), probably based on the individual’s physical makeup and health [1–3]. If these manifestations become excessive, anxiety can manifest negatively, become pathological, and need treatment [4]. Anxiety can range from unnoticeable to extremely high levels, capable of altering the individual’s functioning in several ways. Some examples of extreme manifestations of anxiety are panic attacks and phobias [5].

The proportion of the world population with anxiety disorders in 2019 was estimated at 3.94% [6]. According to the same authors, in 2019, 8.79% of the population in Portugal was estimated to have an anxiety disorder, a condition increasingly more present in today’s society due to the stressful pace of life and high expectations individuals set for themselves. Therefore, there should be assessment tools that allow for a detailed and rigorous assessment of this phenomenon so that healthcare workers can reliably characterise it [7].

In a review of anxiety assessment tools, the following instruments were found that are validated for Portugal: (a) State-Trait Anxiety Inventory (STAI), developed by Spielberger et al. [1], which was validated for Portugal by Silva and Campos [8]; (b) Hospital Anxiety and Depression Scale (HADS), developed by Zigmond and Snaith [9], which was validated for Portugal by Pais-Ribeiro, Silva and Ferreira [10]; (c) Beck’s Anxiety Inventory, developed by Beck, Epstein and Brown [11] and translated and adapted to the Portuguese population by Quintão, Delgado and Prieto [12]; and (d) Zung’s Self-Assessment Scale of Anxiety, developed by Zung [13] and validated in a sample of the Portuguese population by Vaz Serra, Ponciano and Relvas [14]. However, these are all self-report measure instruments.

This psychometric study arose from establishing that only a few clinician-rated measures of anxiety exist that are validated for the Portuguese population. For this reason, we aimed to translate and validate a clinician-rated measure of anxiety for the Portuguese population. Conducting validation studies of assessment tools for different countries and contexts is crucial because multiple validations allow for a more robust assessment of the psychometric properties of the tools and enable researchers to compare the psychometric properties of different tools that assess the same construct.

Thus, the present study aimed to translate and culturally adapt the Hamilton Anxiety Scale for the Portuguese population and assess its psychometric properties in a sample of adult people with mental health disorders.

## Methods

### Design

This psychometric study was carried out using a non-probability convenience sample. In the first phase, the assessment tool was translated and culturally adapted to European Portuguese, following the guidelines of the International Test Commission [15]. In the second phase, the psychometric properties of the assessment tool were assessed using the IBM SPSS® software version 27.

### Translation and cultural adaptation

Two independent native speakers of European Portuguese – a nurse and a professional translator – with native-like command of English (and one of them having extensive knowledge of the theoretical construct of the assessment tool) were involved in the translation process from English into European Portuguese [15]. After the two translations (T1 and T2) were ready, the research team compared the two renditions and reached a consensus version (TS).

Once this stage was concluded, the consensus version was back-translated into English, the original language of the assessment tool, by two other independent translators with native-like command of European Portuguese and English and no knowledge of the original scale. These back-translations resulted in two versions which we labelled B1 and B2.

Finally, the seven professionals who participated in this process analysed and reviewed all the renditions (T1, T2, TS, B1, B2). Those professionals were nursing professors with experience in methodological studies, mental health nurses, nurses with native-like command of English, and a translator. We chose this group of persons following the recommendations of Beaton, Bombardier and Guillemin [16], who advocate that the cross-cultural equivalence of an assessment tool’s translation and adaptation should be validated by a group including at least researchers with experience in methodological studies, health professionals, and language professionals, including translators.

After consensus was reached on the final version of the assessment tool and the analysis phase was completed, a data collection tool pretest was conducted. The pretest aimed to evaluate: if the terms in the Hamilton Anxiety Scale were understandable and free from misunderstandings, if the format of the questions in the data collection tool allowed the collection of the intended information; and if the data collection tool was not too long and did not cause disinterest. The pretest was conducted with 16 adults diagnosed with anxiety and/or depressive disorders, recruited from the Psychiatry Department of the Hospital Center of Vila Nova de Gaia / Espinho (CHVNG/E) and the Magalhães Lemos Hospital. This pretest was conducted by five selected mental health nurses working in the abovementioned institutions to ensure that the questions were clear and objectively assessed what the research team intended [17]. According to Fortin et al. [18], in the pretest, the sample should consist of 10 to 30 subjects.

### Evaluation of the psychometric properties

#### Participants and settings

The study was conducted in a Psychiatry Department in the Northern region of Portugal, including inpatients, day hospital patients, and outpatients.

The target population was patients of that department who met the following inclusion criteria: (a) age 18 to 64 years and (b) with a diagnosis of anxiety or depressive disorders. However, patients were asked to participate in the study only if the diagnosis of anxiety or depressive disorder was registered and currently active in the electronic health records.

Patients with either (a) moderate or severe dementia or moderate to severe intellectual impairment; (b) a confusional state; (c) psychomotor agitation; (d) hostility or latent aggressiveness; (e) mutism or impaired expressive communication; or (f) significant hearing loss that prevented the interview were excluded from this study. The researcher assessed exclusion criteria (b) to (f) by observing the patients’ behaviours during the interview; the exclusion criterion (a) was previously consulted in the electronic health records, so patients were asked to participate in the study only if they did not present that exclusion criterion. These conditions were excluded because, according to Hamilton [19], anxiety is found in states such as dementia and schizophrenia to a greater or lesser degree. Still, it should be clear that the scale is not intended to be administered in these conditions. Moreover, when validating the original version of the tool, the same author defined it as one to assess anxiety in adults, thus justifying the choice of the target population. We included people with depressive disorders because, although the original scale developed by Max Hamilton was intended for assessing patients with anxiety disorders as the primary diagnosis, international studies, namely in Denmark [20] and Spain [21], conducted to assess the psychometric properties of Hamilton Anxiety Scale also included patients with that diagnosis with good results.

The sample size was determined by the number of participants needed to conduct exploratory and confirmatory factor analyses. According to Tabachnick and Fidell [22], at least 10 individuals are required for each item to conduct exploratory factor analysis (EFA). According to Hu and Bentler [23], the minimum sample size for conducting an EFA is five individuals per variable. Therefore, since the Hamilton Anxiety Scale comprises 14 items, the minimum number of participants to be included in the sample was 140.

#### Data collection procedures

A questionnaire was used for sociodemographic and clinical evaluation, identifying data such as age, gender, education, marital status, and pathology presented by the patient. We used two assessment tools for assessing anxiety.

One of the assessment tools was the Hamilton Anxiety Scale, which is the one being validated in the current study, a clinician-rated instrument composed of 14 items (that encompass somatic and psychic factors) that are rated in terms of intensity from 0 (not present) to 4 (very severe). The patient’s anxiety level is identified after the ratings of all items are summed up. The maximum possible score is 56. An overall rating of less than 17 indicates mild anxiety, from 18 to 24 shows moderate anxiety, and 25 or more reveals moderate to severe anxiety [24].

Previous psychometric explorations of the Hamilton Anxiety Scale suggest an optimal two-factor structure [19, 25], while others suggest an optimal three-factor structure [26]. Common across both two- and three-factor structures is the fact that somatic symptoms of anxiety form a separate factor, while anxiety and depressive symptoms may be represented by either a unitary factor (in two-factor models) or separate factors (in three-factor models) [27]. In the original version of the Hamilton Anxiety Scale [19], the “psychic anxiety” factor included the following items: anxious mood, tension, fears, insomnia, intellectual (cognitive changes), depressed mood, and behaviour during the interview; on the other hand, the “somatic anxiety” factor included the following items: general somatic symptoms (muscular), general somatic symptoms (sensory); cardiovascular symptoms, respiratory symptoms, gastrointestinal symptoms, genito-urinary symptoms, and autonomic symptoms. Regarding the Hamilton Anxiety Scale reliability, its internal consistency (Cronbach’s alpha) ranged from 0.77 to 0.92 in previous studies [19, 28].

The other assessment tool was an anxiety self-report assessment instrument known as STAI. That assessment tool consists of two self-completion subscales, one assessing anxiety at the moment (state) and the other assessing the tendency to feel anxious (trait). The European Portuguese version of the STAI was used to assess its convergent validity with the Hamilton Anxiety Scale in the target population [8]. This assessment tool was chosen because it is the gold standard for assessing anxiety and is widely used in several countries and international studies. In Portugal, three studies have been conducted using the STAI [29]. The first study is the most comprehensive, involving different population groups, from students to adults working in their profession aged 19 to 39 years [8]. The internal consistency values were good for both male (0.88) and females (0.93) [8].

We did not consider any of the instruments to assess depressive symptoms exclusively since this is not the core construct of the Hamilton Anxiety Scale. However, some items of the Hamilton Anxiety Scale assess depressive symptoms since, as described in the literature, people with anxiety disorders usually also present depressive symptoms [30].

The data collection tool was administered from March to July 2021. The privacy of each of the 140 participants was respected by administering the data collection tool individually.

During the same period, an independent researcher (a mental health nurse) administered the Hamilton Anxiety Scale to 61 of the 140 participants immediately after the study’s first author (also a mental health nurse) administered the scale. The raters had no specific training in scoring the Hamilton Anxiety Scale. However, because they were mental health nurses, they were professionally trained to administer and score mental health screening tools. The Hamilton Anxiety Scale was administered in two consecutive moments but in separate rooms aiming to analyse its inter-rater reliability using ICC. To ensure that the half width of a 95% two-sided confidence interval for *ρ* was no greater than *ω* with 50% assurance probability, a minimum number of 61 participants was required to detect the ICC for inter-rater reliability [31].

#### Data analysis

We conducted frequency analysis to obtain the sociodemographic and clinical characteristics, and we calculated means and standard deviations (SD) to examine the central tendency and dispersion of the data.

To ascertain the underlying dimensionality of the Hamilton Anxiety Scale’s construct, it was first necessary to evaluate whether the data met the assumptions for this analysis, which was verified using the Kaiser–Meyer–Olkin (KMO) test and Bartlett’s sphericity test. The underlying factors within the construct were determined by exploratory factor analysis using the principal axis factoring estimation method. Varimax rotation [32], which is the same method that has been used in international validation studies of the same assessment tool [21] and the validation of the original tool [19], was used. This analysis was performed to verify if, as in the validation of the original version, the best factor solution is one with two factors (i.e., psychic anxiety and somatic anxiety), and to assess the total variance explained by the factors and to assess the total variance explained by the factors [19].

Then, the Hamilton Anxiety Scale’s factor structure was analysed using the CFA to assess whether the same factors as the original scale version fit the data from our translated version. That analysis was performed using the IBM SPSS Amos® software.

Considering that, according to the results of the Kolmogorov–Smirnov test, data were normally distributed, convergent validity was assessed using Pearson’s correlation coefficient, which analysed the correlations between our new translated Hamilton Anxiety Scale and the State-Trait Anxiety Inventory (STAI) subscales.

Finally, reliability was analysed using Cronbach’s alpha to assess internal consistency and by analysing inter-rater reliability using the intraclass correlation coefficient (ICC). Statistical significance was considered to exist whenever *p* < 0.05.

### Ethical considerations

The study was approved by the Ethics Committee of the Hospital Center of Vila Nova de Gaia / Espinho (CHVNG/E) – reference I.HU9_CES:28/2021.

All the participants agreed to take part in the research voluntarily after learning thoroughly about the study and being guaranteed the anonymity and confidentiality of the answers, as expressed in the free and informed consent form following the Declaration of Helsinki [33] and the Oviedo Convention [34], which they all read and signed.

## Results

### Translation and cultural adaptation

Two independent translations were obtained in the first step of the adaptation process. Overall, the two translations were quite similar. However, there were few specific differences among the Hamilton Anxiety Scale items.

After obtaining those two versions, a consensus version was developed by the research team, who analysed both versions and reached a consensus on the different terms. Concerning severity ratings, the words “absent”, “severe”, and “very severe” were chosen because they appeared to be widely used terms in assessment tools in the field. Thus, all items were standardised in a single version of the scale.

The consensus version obtained was then back-translated into English. Once again, this version was very similar to the original, with slightly different terms but similar meanings. The back-translation did not present significant divergences from the original version of the Hamilton Anxiety Scale. After this process, the seven professionals who participated in the translation and adaptation process of the Hamilton Anxiety Scale met and endorsed the final consensus version of the tool.

Before evaluating the psychometric properties, five mental health nurses conducted a pretest. Their feedback was that the scale took approximately 10 to 15 min to complete and was easily understood by the rater. However, mental health nurses reported that some items included many aspects to evaluate, which may lead to evaluation bias. Essentially, the assessment tool was considered to be useful. Nonetheless, specifying severity levels within the range of absent to very severe was considered, by mental health nurses, not to be an easy task.

### Evaluation of the psychometric properties

#### Respondents’ characteristics

The study included 140 adult patients aged 18 to 64 years, followed up in settings of a Psychiatry Department (Inpatient—14.30%; Day Hospital—32.10% and Outpatient—53.60%), with anxiety disorders, depressive disorders, or both.

The mean age of the respondents was 46.24 years (SD = 13.49 years, range 18 to 65), and 79.30% were female, mostly married (50.70%). Only 15.80% of the sample had completed higher education. The sociodemographic and clinical characteristics of the respondents are shown in Table 1.

**Table 1:** Sociodemographic and clinical characteristics of the sample

Variables	*n*	%
Gender		
Male	29	20.70
Female	111	79.30
Marital Status		
Single	32	22.90
Married or in a consensual union	71	50.70
Divorced or de facto separated	27	19.30
Widower or widow	10	7.10
Education		
4^th^ year	21	15.00
6^th^ year	19	13.60
9^th^ year	29	20.70
12^th^ year	49	35.00
Degree (Bachelor, College, Licentiate)	18	12.90
Master’s Degree	4	2.90
Diagnosis		
Anxiety Disorder	48	34.30
Depressive Disorder	38	27.10
Anxiety and Depressive Disorder	54	38.60

#### Psychometric properties

The KMO value obtained was 0.91, and Bartlett’s test was significant (χ^2^(91) = 1053.486; *p* < 0.01). As such, these values were considered acceptable for factor analysis.

An exploratory factor analysis was performed with varimax rotation. The extraction was based on eigenvalues greater than 1. The analysis resulted in two factors explaining 57.20% of the variance.

The CFA results did not indicate that the model effectively fits the data. Therefore, we tried to identify the origin of those modest results and verified that they were mainly due to items 1 and 2 and 9 and 10. Thus, a new CFA was carried out with correlations among error terms for items 1 and 2 and between error terms for items 9 and 10.

Table 2 shows the results obtained from the CFA performed with correlations among error terms for those two subsets of items.

**Table 2:** Results of the Confirmatory Factor Analysis (CFA)

Parameters	Results
CMIN/DF	1.29
Bentler’s Comparative Fit Index (CFI)	0.98
Root Mean Square Error of Approximation (RMSEA)	0.05
Root Mean Square Residual (RMR)	0.09
Goodness of Fit Index (GFI)	0.91

**Fig. 1:**
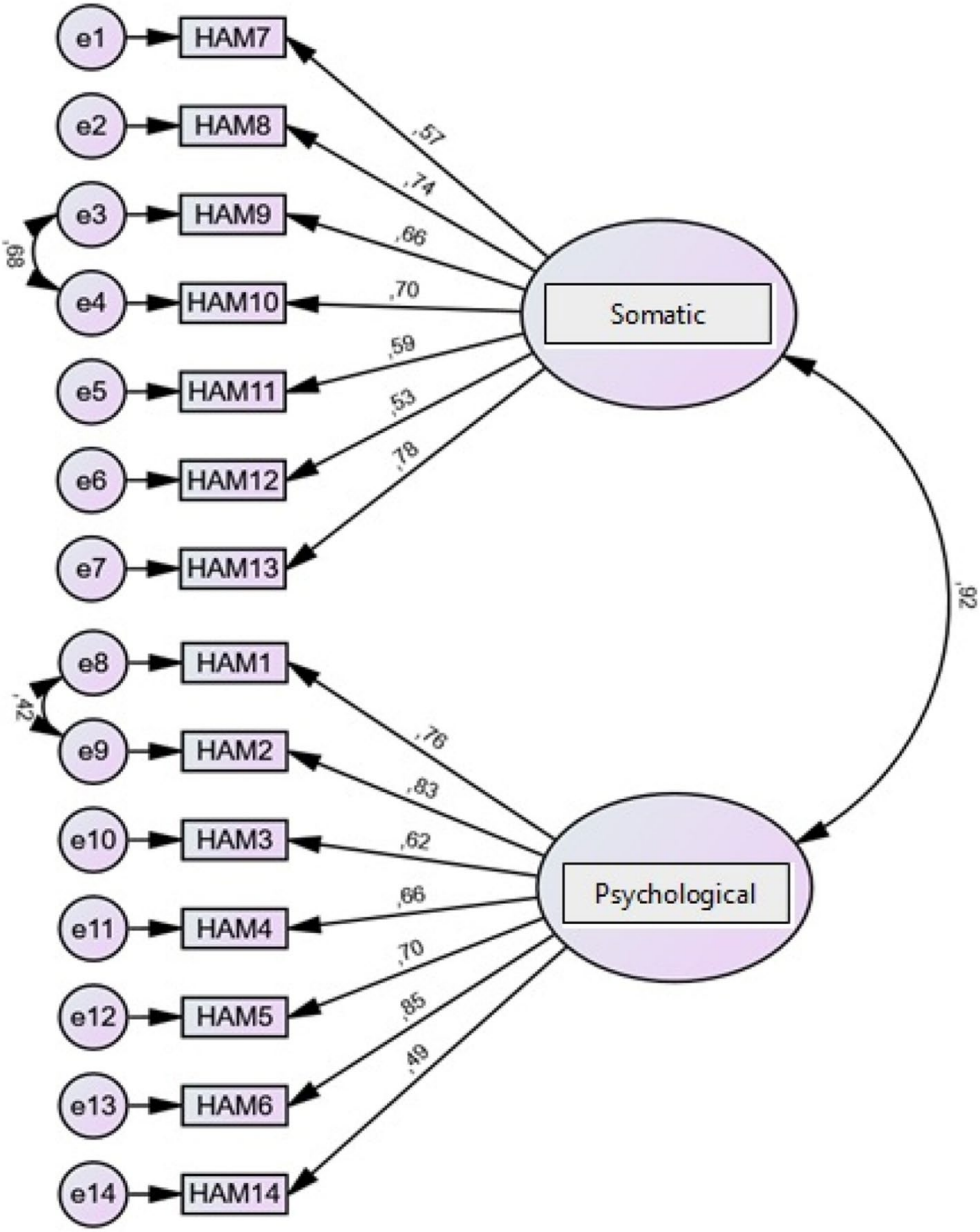
Confirmatory factor analysis (CFA)

Figure 1 below shows the final CFA model performed with correlations among error terms for the two subsets of items.

Convergent validity between the Hamilton Anxiety Scale and the STAI subscales was assessed using Pearson’s correlation coefficient. Pearson’s correlation coefficient was also calculated between the STAI subscales and the two factors of the Hamilton Anxiety Scale, namely “Psychic Anxiety” and “Somatic Anxiety” (Table 3). The results showed that the overall scale and subfactors show a moderate correlation with trait anxiety, as measured with the STAI. However, there are no significant associations with the STAI “State Anxiety” subscale.

**Table 3:** Results of the Pearson correlation between the Hamilton Anxiety Scale, the Hamilton Anxiety Scale factors, and the State-Trait Anxiety Inventory

	Hamilton Anxiety Scale		Hamilton Psychic Anxiety Factor		Hamilton Somatic Anxiety Factor	
	*r*	*p*	*r*	*p*	*r*	*p*
STAI Anxiety State Subscale	.09	.28	.08	.32	.09	.29
STAI Anxiety Trait Subscale	.31^*^	.00	.29^*^	.00	.30^*^	.00

*r* – Pearson correlation coefficient

*p* – level of statistical significance

^*^p < 0.01

Reliability was analysed by internal consistency through Cronbach’s alpha, with a value of 0.92. This test was performed for the Hamilton Anxiety Scale and for each of its factor, namely the “Somatic Anxiety” factor (Cronbach’s alpha = 0.85) and the “Psychic Anxiety” factor (Cronbach’s alpha = 0.87).

The inter-rater reliability analysis was performed using the ICC, item by item, and then for the total score of the Hamilton Anxiety Scale. The ICC value obtained for the total score of the scale was 0.91.

## Discussion

The main aim of this study was to translate, culturally adapt and assess the psychometric properties of the Hamilton Anxiety Scale into European Portuguese in a sample of adults with mental health disorders. According to the findings, the validity and reliability of the assessment tool were supported; however, the its use should be carefully considered.

As for the participants’ sociodemographic characteristics, the mean age was 46.24 years, and most were female (79.30%). Compared to the statistical data presented by the literature, worldwide, as with depression, anxiety disorders are more common among women (4.70% compared to 2.80% in men) [6]. Of the participants in this study, 34.30% had an anxiety disorder, 27.10% had a depressive disorder, and 38.60% had both disorders at the same time, as is commonly described in the literature. According to the World Health Organization (WHO) [35], many people experience both conditions (anxiety disorder and depressive disorder) simultaneously, a fact that was confirmed in this study after analysing the sociodemographic characteristics of the sample, as revealed earlier; among others, this was one of the criteria for defining the target population of this study.

In the EFA with varimax rotation performed, in line with the validation procedures of the original version of the Hamilton Anxiety Scale and other international validations [19, 21], the total variance explained by two factors was 57.20%. According to Hair, Anderson and Black [36], the acceptable variance explained in factor analysis for a construct to be valid is at least 60%, so the value found is below the reference value. However, given the proximity to the recommended minimum of 60%, this value must be confirmed in subsequent validation studies. Nonetheless, the obtained value was more satisfactory than in the original version of the Hamilton Anxiety Scale, in which the total variance explained by two factors was only 45% [19].

The CFA was performed with correlations among error terms for two subsets of items, items 1 and 2, which assessed anxious mood and tension, and items 9 and 10, which assessed cardiovascular and respiratory symptoms, respectively. According to the literature, there are reports of the interconnection between these items. Whartin [37] described that patients with generalised anxiety disorder have worries or feelings of anticipation of the worst about various situations and are therefore constantly tense, demonstrating that the items 1 and 2 are closely related. On the other hand, items 9 and 10 are also mentioned in some studies as strongly associated. Anxiety states often encompass respiratory symptoms, such as dyspnoea, and other symptoms, including chest tightness, palpitations, and feeling faint, i.e., cardiovascular symptoms, are common at the same time [38].

From the CFA with correlations among error terms for two subsets of items, we observed that the factor loadings for each of the items were higher than 0.49, which indicates they present a moderate to high factor loading since the minimum acceptable value of a factor loading is higher than 0.45 [22]. In this sense, it is possible to state that there is a cluster of items that allows assessing somatic anxiety and that there is another cluster of items that allows assessing psychic anxiety. If one factor assesses the psychic part and another assesses the somatic part, the sum of the two will allow a comprehensive assessment of anxiety.

In the CFA we obtained the value CMIN/DF = 1.29, which seems to be positive since, according to Kline [39], for the CMIN/DF to present acceptable values it should ideally be less than 3, indicating an acceptable fit between the hypothetical model and the sample data. In this study, we obtained a CFI = 0.98, which seems to be a positive result since CFI values higher than or equal to 0.90 are considered acceptable [39]. In turn, the RMSEA obtained was 0.05, which also seems to be a positive result, since the RMSEA 0.05 to 0.08 are deemed acceptable [40]. For the RMR the value obtained was 0.09, with values less than or equal to 0.10 being considered acceptable [36]. Finally, the value obtained for the GFI was 0.91, and according to Hair et al. [36], the GFI has values considered acceptable when equal to or higher than 0.85.

Concerning convergent validity, the obtained values showed a positive correlation between the total score of the Hamilton Anxiety Scale and the STAI “Anxiety Trait” subscale, revealing convergent validity between them. However, no statistically significant correlation was found between the Hamilton Anxiety Scale and the STAI “Anxiety State” subscale. The convergent validity between the somatic and psychic factors of the Hamilton Anxiety Scale and the STAI was also analysed, and the findings were similar; that is, both factors, the somatic and the psychic, showed a statistically significant correlation with the STAI “Anxiety Trait” subscale and no statistically significant correlation with the STAI “Anxiety State” subscale.

This fact can be partially explained by the conclusions found by Maier et al. [28] in a study in which the Hamilton Anxiety Scale was administered to two samples, the first composed of 97 patients with anxiety disorders and the second composed of 101 patients with depressive disorders. They identified [28] a high degree of overlap between the anxiolytic effects measured by the Hamilton Anxiety Scale change scores and the antidepressant effects, and also that the Hamilton Anxiety Scale was unable to detect anxiety states in the sample of depressed patients [28].

These results may also be related to the differences between a self-report measure and a clinician-rated measure since the patient’s perception may be different from the healthcare worker’s assessment. Thus, on the one hand, healthcare workers may overvalue or undervalue some aspects differently from the patients; on the other hand, patients may minimise or maximise their complaints to healthcare workers, and may not respond as freely in an interview as when filling out an assessment tool [41]. Finally, another potential explanation for the non-significant correlation between the total scores of the Hamilton Anxiety Scale and the STAI “State Anxiety” subscale may be the time period over which the assessment is made, as the STAI “State Anxiety” subscale invites the respondents to consider just the present moment.

In this study, Cronbach’s alpha found was 0.92. According to the literature, a Cronbach’s alpha value ≥ 0.70 is considered adequate [42], and a value ≥ 0.90 is considered excellent [43]. Therefore, the result obtained demonstrates an excellent internal consistency of the Hamilton Anxiety Scale.

A total ICC of 0.91 for a 95% CI was obtained. The inter-rater reliability is considered very good if the coefficient is higher than 0.90, good if it is 0.71 to 0.90, average if it is 0.31 to 0.50, and poor or null if it is lower than 0.31 [31, 44, 45]. Thus, we can state the scale presented a very good inter-rater reliability.

According to the literature, when comparing to validation studies carried out in other countries for the same assessment tool, in a Spanish study carried out by Lobo et al. [21] the Cronbach’s α of the Hamilton Anxiety Scale was 0.89, while our findings indicated a higher internal consistency (Cronbach’s α = 0.92). Concerning the inter-rater reliability, Maier et al. [28], in a study conducted in Germany, found a lower ICC (= 0.74). However, the study carried out by Lobo et al. [21] found an ICC which is in line (= 0.92) with the one that was found in our study (= 0.91). It should be noted that only international articles validating the Hamilton Anxiety Scale in adults with anxiety or depressive disorders or both were used to compare the psychometric data obtained in our study. Other anxiety assessment tools validated for the Portuguese population have the following psychometric properties: (a) Hospital Anxiety and Depression Scale (HADS) – Cronbach’s alpha of the “Anxiety” subscale = 0.76 [10]; (b) Beck Inventory – Cronbach’s alpha = 0.92; ICC = 0.75 [46]. Comparing these results with those obtained in this study (Cronbach’s alpha = 0.92 and ICC = 0.91), we can conclude that, in terms of internal consistency, the results were similar to those of the Beck Inventory and slightly better than those of the HADS “Anxiety” subscale.

### Limitations

The results should be analysed considering the following limitations: the sample was composed of patients from the same department and the same hospital. Thus, future studies should also include patients who are followed up in other hospitals or departments. It could be an added value to carry out this study at the primary healthcare level since many patients diagnosed with anxiety and depressive disorders are only followed up by their general practitioner, and only the most severe cases are transferred to hospitals. In addition, it would be important to use a probabilistic sampling technique, so that the sample would be more representative, and to assess the temporal stability of the scale through test–retest, although it is known that anxiety is a rapidly changing construct; therefore, the assessment at different moments could lead to different results, not because of lack of stability of the scale, but because there was an actual change in the person’s anxiety level, which could cause bias in the results.

## Conclusions

The evaluation of the psychometric properties corroborated the appropriateness of the Hamilton Anxiety Scale for use in clinical practice in patients with anxiety and/or depressive disorders. However, given that the convergent validity between the Hamilton Anxiety Scale and the STAI “State Anxiety” subscale was not statistically significant, it should be carefully used until further comparative studies between these two assessment tools are carried out. If the lack of convergent validity with the STAI “State Anxiety” is confirmed, it will be necessary to carry out studies to clarify whether the Hamilton Anxiety Scale shows convergent validity when compared to other anxiety assessment tools validated for the Portuguese population and whether this is a potential path to follow in future research.

The Hamilton Anxiety Scale is a tool of easy use and applicability in clinical settings by healthcare workers, especially useful for mental health professionals. It may be administered in patients with mental health disorders, specifically anxiety and/or depressive disorders. Thus, it is important to continue and replicate this type of study, both in Portugal and in other countries, since several validation studies of the Hamilton Anxiety Scale will allow for a more robust evaluation of its psychometric properties, allowing researchers in this field to compare the psychometric properties of different assessment tools that assess the same construct according to the countries and/or contexts in which they were validated.

## Data Availability

The datasets used and/or analysed during the current study are available from the corresponding author on reasonable request.
